# Effect of Wet Mixing on Properties of Radial-Orientation Basalt Fiber-Reinforced Rubber Compounds

**DOI:** 10.3390/polym14204422

**Published:** 2022-10-19

**Authors:** Benhui Yu, Jing Wang, Kongshuo Wang, Deshang Han, Jianbin Ren, Dewei Zhang, Chuansheng Wang

**Affiliations:** 1 College of Electromechanical Engineering, Qingdao University of Science and Technology, Qingdao 266061, China; 2 Shandong Provincial Key Laboratory of Polymer Material Advanced Manufactorings Technology, Qingdao University of Science and Technology, Qingdao 266061, China

**Keywords:** basalt fibers, radial orientation, qualitative characterization, wet mixing, delay

## Abstract

The effects of wet mixing and traditional mixing on the properties of radial-orientation basalt fiber-reinforced rubber products were studied through experiments. The results show that compared with traditional mixing, the basalt fibers under the wet mixing conditions can more effectively enhance the physical and mechanical properties of composites. The properties of the composites, such as carbon black dispersion, filler dispersion, rolling resistance and wet-sliding resistance, were the best after the latex and carbon black were premixed and then mixed by a mixer. Through extrusion experiments with the developed short-fiber radial-orientation die, it can be found that the fluidity of composites after extrusion is enhanced. Through analysis utilizing an electron microscope, it is shown that when the BFs added with KH550 (3-Aminopropyltriethoxysilane) were modified by KH560 ((3-Glycidyloxypropyl)trimethoxysilane), the interface layers of BF (basalt fiber)–KH560–NR and BF–KH550–NR were formed, which improves the adhesion between BFs and the rubber matrix. Qualitative characterization experiments on the orientation direction of the vulcanized composites were carried out through the experiments; that is, the qualitative characterization experiments on the segmented cutting and vulcanization of the composites in the radial direction showed that the short-fiber radial-orientation die could greatly improve the radial orientation degree of the short fibers in the radial direction. After adding KH560, the performance of the composites reinforced by the short fibers was improved to a certain extent compared with those without KH560. By adding DZ (N,N-Dicyclohexyl-2-benzothiazolsulfene amide) and CTP(cytidine triphosphate disodium) into the vulcanization system, the curing process of compounds in mixing and extrusion was delayed and the scorching resistance of short-fiber-reinforced composites was enhanced. Under the same conditions, the properties of the compounds after extrusion were greatly improved compared with those without extrusion.

## 1. Introduction

Short fiber–rubber composite (SFRC) is a type of polymer material with high performance, which has the rigidity of short fibers and the high elasticity of rubber, and gives its products a series of excellent properties such as high modulus, high hardness, tear resistance, puncture resistance, cutting resistance, low-pressure shrinkage deformation, load fatigue resistance, low heat generation, swelling resistance and creep resistance [1,2,3,4,5,6,7,8,9,10,11,12,13,14,15]. Stephan Günzel [2] et al. found that short glass fiber-reinforced polyamides are increasingly used in automotive applications. Concepts and models are needed that enable the prediction of structural durability. The fiber orientation and the content of moisture have a major influence on the fracture mechanical properties and on the damage mechanisms. A series of short aramid fiber-reinforced ethylene–propylene–diene terpolymer (EPDM)/acrylonitrile–butadiene rubber (NBR) composites with designed fiber orientation angle (β) between the short-fiber orientation and the ablative surface have been prepared in the work of Guoxin Gao et al. [3]. The influence of β on the thermal degradation and ablation resistance properties of composites under the oxyacetylene flame is carefully discussed. With an increasing in β, the porosity of the char layer is reduced, whereas its hardness is improved. In the aerospace field, fiber-reinforced composites have become an important thermal insulation functional material. In the work by Winifred Obande et al., the viscoelastic and impact behaviors of glass fiber-reinforced composites, containing Elium^®^ and a commercial epoxy matrix, were evaluated. To complement observations from both characterization techniques, the fracture characteristics of both materials were also investigated by scanning electron microscopy. M. Hassani Niaki et al. [15] describe the investigation of the synthesis and improvement of the mechanical properties of quaternary epoxy-based polymer concrete (PC) using basalt fiber and clay nanoparticles. Arumugam [16] found that fiber-reinforced composite materials used in aircraft as structural materials have high rigidity and strength; however, if the short fibers are oriented in a certain direction, the comprehensive properties in that direction can be greatly enhanced.

**Figure 1:**
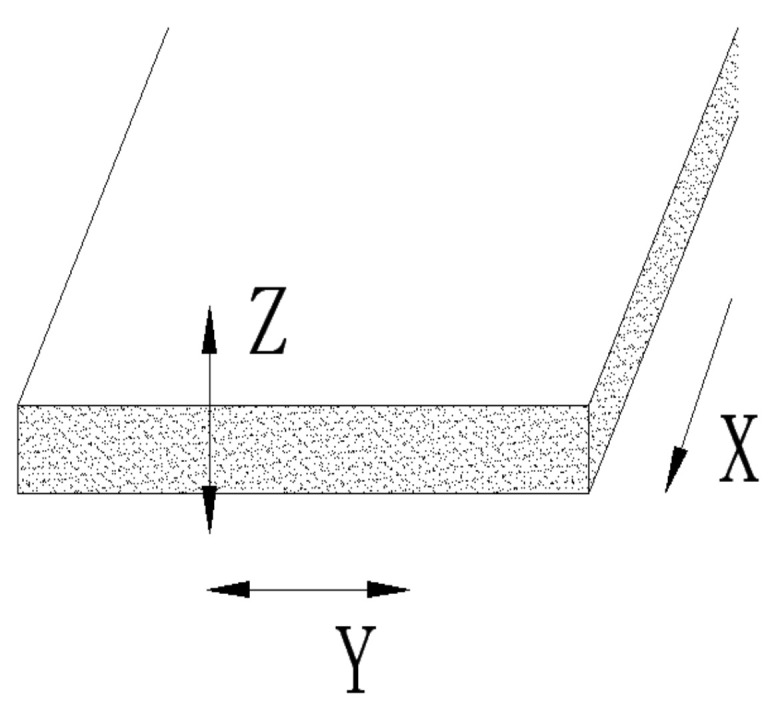
Schematic image of short-fiber orientation definition.

Fiber-reinforced rubber products will have anisotropy. For example, this anisotropy will have a significant impact on the mechanical properties, ablation properties and shrinkage rate of the EPDM insulation layer [3]. Figure 1 is a schematic diagram of the short-fiber orientation definition. At present, there are many studies on the hybrid of polypropylene fiber and steel fiber, but the cost of steel fiber materials is high, and there are problems such as corrosion and conductivity. More and more experts and scholars turn to the research of PVA fiber and basalt fiber [17]. Basalt fiber is a kind of continuous fiber drawn from natural basalt. It is a high-performance inorganic non-metallic fiber material.

Basalt continuous fiber not only has high strength, but also has many advantages such as electrical insulation, corrosion resistance and high-temperature resistance. In addition, compared with most industrial fibers, basalt fibers have better anti-aging performance [18]. Basalt fiber is easy to be oriented and dispersed, but its surface is smooth and its adhesion with rubber and plastic matrices is poor. Therefore, the fiber surface needs to be treated.

Although the traditional method can mix the rubber with excellent performance to a certain extent, and the production capacity can be guaranteed, it still has the problems of high unit energy consumption, poor production environment, difficult-to-control process conditions, and uneven dispersion of filler additives such as short fibers [19,20,21].

Therefore, there is an urgent need to innovate the mixing method and theory. The wet mixing technology, i.e., the lotion blending method, produces rubber-based composite materials [22]. In the paper “preparation mechanism and experimental research of carbon fiber reinforced natural latex composite materials” [23], the modification mechanism of dopamine and a silane coupling agent on carbon fiber was analyzed on the basis of natural latex.

The effects of carbon fiber addition, different concentrations of dopamine, modification time, different silane coupling agents and addition amount on the properties of carbon fiber/rubber composites were studied. However, for basalt fibers with high rigidity, inert surfaces and poor adhesion, it is an important research direction to take necessary measures to improve the surface adhesion at this stage, and the dispersion of basalt-fiber filler in traditional internal mixer mixing needs to be improved. Generally, the processing performance of rubber compound is obviously improved after mixing and extrusion by extruder and vulcanization. The direction of short fibers in the rubber is oriented in the radial direction. The extruder-head configuration is a common method. However, the qualitative characterization of the physical and mechanical properties of short fibers in the radial direction is also an important problem solved by existing technology. The existing test methods can only test the axial and circumferential directions. The qualitative characterization of fibers in the radial direction needs to be along the radial direction of the rubber, so it is difficult to test.

## 2. Materials and Methods

### 2.1. Raw Material

Natural rubber, GB 1, produced in Vietnam; BR2000, made in Thailand, chopped basalt fibers (BFs), KH560 pretreatment, lengths: 3 mm, 9 mm, diameter 27 um, Hebei Baoding synthetic material factory; silane coupling agent KH560 (Purity: 98%), Shanghai Yaohua chemical plant; N234, Jiangxi Black Cat Carbon Black Co., Ltd., Jingdezhen, China; sulfur, Chaoyang Tianming Industry and Trade Co., Ltd., Beijing, China; ZnO, Hebei Shijiazhuang Zinc Oxide Factory, Shijiazhuang, China; SAD, Fengyi Grease Technology (Tianjin, China) Co., Ltd., China; anti-aging agent 4020, Shandong Shangshun Chemical Co., Ltd., Weifang, China; DZ, Shandong Shangshun Chemical Co., Ltd., Weifang, China. Other materials were provided by the cooperative enterprise Qingdao Railway Rubber Factory.

Other materials: silicon dioxide, Si69, NS, RD, microcrystalline wax, CTP, natural latex (60% by mass).

### 2.2. Experimental Equipment

Open mill, X (s) k-160, Shanghai Rubber Machinery Factory, Shanghai, China; wear tester, GT-2012-D, High Speed Railway Technology Co., Ltd., Taizhong, China; UM-2050 Mooney viscometer, Youken Technology Co., Ltd., Taipei, China; scanning electron microscope, JSM-6700, Japan Electronics Corporation, Tokyo, Japan; RPA rubber processing analyzer, RPA2000, USA Alpha; hardness tester (Shao A), LX-A, Shanghai Liuling Instrument Factory, Shanghai, China; universal tensile testing machine, 3366, Instron Company, Boston, MA, USA; DIN abrasion testing machine, GT-7012-D, High Speed Rail Testing Instrument Company, Taizhong, China.; DisperGRADER Dispersion Meter, American Alpha Company, Akron, OH, USA; LEXT OLS5000 3D laser measuring microscope, Osaka, Japan; dynamic thermomechanical analyzer, EPLEXOR-150N, Gabo Qualimeter Testanlagen GmbH, Ahlden, Germany; M-2000-AN, GOTECH TESTING MACHINES Co., Ltd., Taizhong, China; flat-panel vulcanizer QLB-400X400X2, Qingdao Yadong Machinery Group Co., Ltd., Qingdao, China.

### 2.3. Preparation Experiments of Short-Fiber-Reinforced Composites

#### 2.3.1. Preparation Experiments and Delay Formulation Mechanism of Different Types of Short-Fiber-Reinforced Composites

In the following experiments, the BFs used were pretreated short fibers (commercially available basalt fibers), which were added with KH550 in the drawing process.

Fiber pretreatment again: we put the original pretreated chopped basalt fiber into a beaker, immersed it in 1.5% by mass of coupling agent KH560 solution, stirred it with a high-speed rotary machine at 80 °C for 1 h, and then put it into an oven for drying at 80 °C for 4 h. The reason for selecting 3 phr BF is that the interfacial layer between rubber and short fibers increases with an increase in the phrs. During the stress action, the stress concentration points toward the interface layer increase. When the stress on the interface layer exceeds the range it can bear, the interface layer will break, so the tensile property and tear resistance will decrease. Three phr short fibers were selected based on the preliminary experiments.

**Figure 2:**
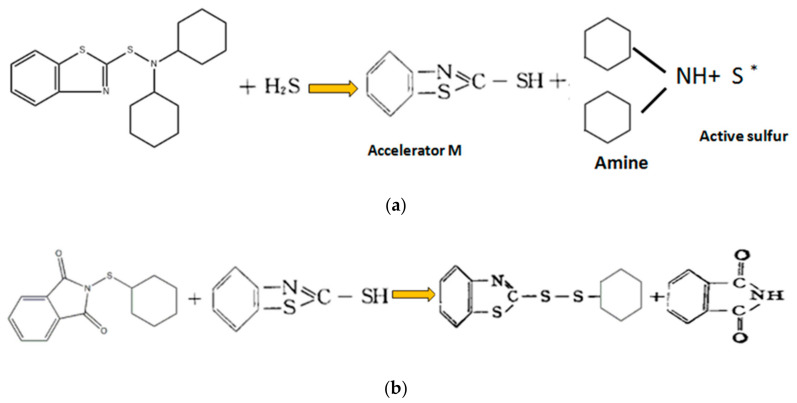
Decomposition mechanism of CTP and disulfonic acid amine accelerants in delayed formulation: (**a**) Reaction of sulfonic amine accelerator DZ during vulcanization; (**b**) Decomposition mechanism of CTP and disulfonic acid amine accelerants in delayed formulation.

As shown in Figure 2, in the experiments, the delayed curing formula was adopted to avoid premature vulcanization of the rubber when the curing system was added to the internal mixer curing process. When the temperature rises to a certain limit, the reaction between rubber and sulfur begins to form hydrogen sulfide, and the decomposition reaction occurs rapidly [24]. Under the combined action of amine and active sulfur, among them, $S^{*}$ represented active sulfur, the resulting polythiobenzothiazole intermediate, i.e., ryrylbenzothiazole, can be vulcanized to produce 2-cyclohexyl disulfide benzothiazole (CDB) and phthalimide, which have no vulcanization effect, thus delaying the vulcanization starting effect and preventing the synergistic effect of amine and polythiobenzothiazole intermediate accelerator M (which is reacted by CTP) to promote the vulcanization, thereby greatly extending the scorching time.

The thioimine CTP with NS structure can easily undergo a nucleophilic substitution reaction at the NS bond and react quickly with accelerator M. the anti-scorching agent CTP has an obvious effect in the sulfur-containing curing system, but the anti-scorching agent CTP has a weak effect if sulfur is not added. When anti-scorching agent CTP and sulfur are used together, the reason why the hyposulfonamide accelerator DZ has a delay effect is that the hyposulfonamide DZ must be converted into a polythiobenzothiazole intermediate, namely ryrylbenzothiazole, before cross-linking occurs. Before vulcanization and a significant reduction in sulfur content, a large amount of the polythiobenzothiazole intermediate accelerator M converted by the sulfosamide accelerator DZ was consumed by CTP.

By adding the vulcanization system combining CTP and DZ, the phenomenon wherein the compounds are prevulcanized by the strong shear heat of the screw (due to the long residence time of the compounds in the screw during the extrusion process of the extruder) can be prevented and, what is more, the screw configuration can be protected.

**Figure 3:**
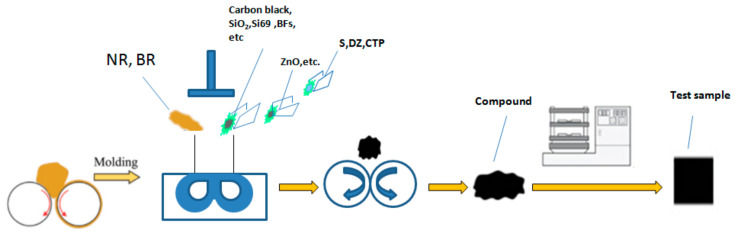
Preparation of different compounds.

As shown in Figure 3 and the Table 1, the following is the preparation method of 1#–7# composites: Short-fiber-reinforced compounds (labeled 1#–7#) were prepared by an internal mixer. The experimental conditions are rotor speed: 70 r/min/40 r/min; cooling water temperature: 60 °C, top bolt pressure: 0.6 MPa; and filling coefficient: 0.6.

1# compound: The fibers in step (a) were 9 mm BFs, which were treated again by KH560 and the compound is the formula of all-steel tires. The mixing method and steps were as follows:

(1) (a) First-stage mixing: we lifted the top bolt, put the NR cut into small pieces into the mixer from the feeding port, closed the top bolt, adjusted the rotor speed to 70 r/min, and mixed for 30 s. We then lifted the top bolt, added 8 phr silica, 22.5 phr carbon black N234, 1 phr Si 69 and 3 phr BF, closed the top bolt, and mixed for 120 s; 70 r/min.We then lifted the top bolt, added 3.5 phr ZnO, 2 phr stearic acid, 1 phr microcrystalline wax, 22.5 phr carbon black, 1.5 phr antioxidant RD, 1.5 phr 4020, 1.6 phr NS and 0.08 phr of plasticizer, closed the top bolt, adjusted the rotor speed to 40 r/min, and mixed for 4 min and 30 s.
  (b) Second-stage mixing: We added 1 phr sulfur to the master batch obtained in step (a), mixed for 1 min, and discharged the compound.
(2) After the mixing was uniform, the sheet was opened, and the obtained rubber was calendered and oriented along the short-fiber direction. The specific method was as follows:

We adjusted the bar pitch of the open mill to 1 mm, and rolled the roll 5 times along the rolling direction of the open mill, then, we adjusted the pitch to 3 mm, and passed the roller 5 times along the rolling direction to obtain the short-fiber-reinforced compound, with most fibers oriented along the rolling direction.

At the end of vulcanization, the performance test should be carried out, and the compound should be stored for 24 h for standby; this is conducive to the uniformity and stability of the compound property and the relaxation of the internal stress of the rubber macromolecule.

The following are the 2#–4# preparation methods of compounds:

(1) The raw materials were proportioned and weighed, and sequentially added to an internal mixer for mixing.

(a) First-stage mixing: we lifted the top bolt, put the raw rubber (NR, BR) cut into small pieces into the mixer from the feeding port, closed the top bolt, adjusted the rotor speed to 70 r/min, and mixed for 30 s.

Then, we lifted the top bolt, added 5 phr silica, 21.5 phr carbon black N234, 0.5 phr Si 69 and 3 phr BF, closed the top bolt, and mixed for 120 s at the speed of 70 r/min.

Then, we lifted the top bolt, added 4 phr zinc oxide, 2 phr stearic acid SA, 1 phr microcrystalline wax, 21.5 phr carbon black, 1 phr antioxidant RD and 2 phr 4020, closed the top bolt, adjusted the rotor speed to 40 r/min, and mixed for 4 min and 30 s.

(b) Second-stage mixing: We added 1 phr sulfur, 1.8 phr DZ and 0.3 phr CTP to the master batch obtained in step (a), and mixed for 1 min and discharged the compounds.

The specific steps of wet mixing before adding to the internal mixer were as follows:

[5#]

(1) Firstly, the natural latex was subjected to wall-breaking treatment, and 850 g natural latex (60% by mass) solution was stirred in a high-speed mixer at 1800 r/min for 8 min;
(2) We took the silane coupling agent KH560, 1.5 g/L, 40 mL, and added it into a 850 g natural latex (60% by mass) solution, and stirred it at 1800 r/min in a high-speed mixer for 1 min;
(3) We cleaned a proper amount of BFs with deionized water (treated with KH550), added BF to the treated natural latex after the water was clear, stirred for 1 min at 1800 r/min under normal temperature, poured it into a glass dish after completion, and dried it in an oven for 6 h; cut into small pieces, the NR/BF masterbatch was prepared.

[6#]

(1) Firstly, the natural latex was subjected to wall-breaking treatment, and 850 g natural latex (60% by mass) solution was stirred in a high-speed mixer at 1800 r/min for 8 min;
(2) We washed 3 phr BF with deionized water (treated with KH550). After the water was clear, we put 3 phr BF into 40 mL of 1.5 g/L KH560 solution with equal concentration, and heated it in water bath for 2 h. The coupling agent KH560 was attached to the surface of the fibers; it was taken out and placed in the oven for 4 h of drying;
(3) We took out the BF after BF drying, added the BF to the treated natural latex, stirred it at 1800 r/min for 1 min under normal temperature, poured it into the glass dish after completion, and dried it in the oven for 6 h; cut into small pieces, the NR/BF masterbatch was prepared.

[7#]

**Figure 4:**
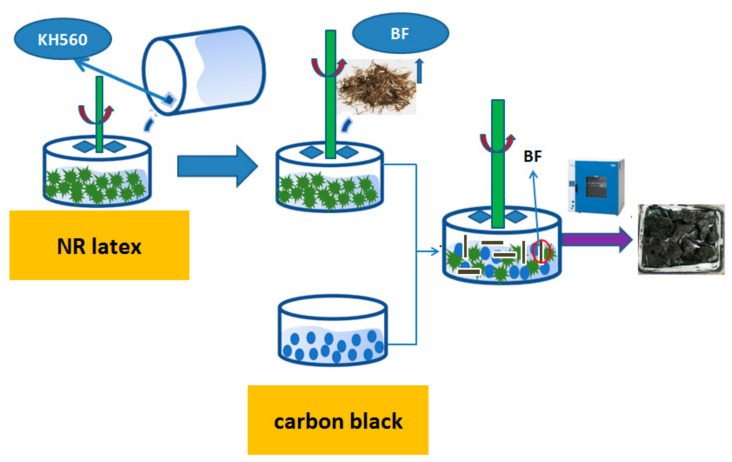
Preparation of rubber and carbon black composites in wet mixing.

As shown in Figure 4,

(1) Firstly, the natural latex was subjected to wall-breaking treatment, and 850 g natural latex (60% by mass) solution was stirred in a high-speed mixer at 1800 r/min for 8 min;
(2) We took silane coupling agent KH560, 1.5 g/L, 40 mL, and added it to the 850 g natural latex (60% by mass) solution, and stirred it at 1800 r/min in a high-speed mixer for 1 min. We then washed a proper number of BFs with deionized water (treated with KH550), added 3 phr BF to the treated natural latex after the water was clear, and stirred at 1800 r/min for 1 min under normal temperature;
(3) We weighed 258 g carbon black (N234) and put it into a beaker, then poured 800 g deionized water. Then, we stirred the carbon black aqueous solution in a stirrer with a stirring speed of 750 r/min and a stirring time of 5 min to fully dissolve the carbon black;
(4) Then, the latex was poured into the carbon black aqueous solution (poured while stirring); the stirring speed was 750 r/min, and the stirring time was 1 min. After completion, it was poured into the glass dish, dried in the oven for 6 h, and then poured into the large tray to be laid and dried to prepare the NR/BF masterbatch.

**Figure 5:**
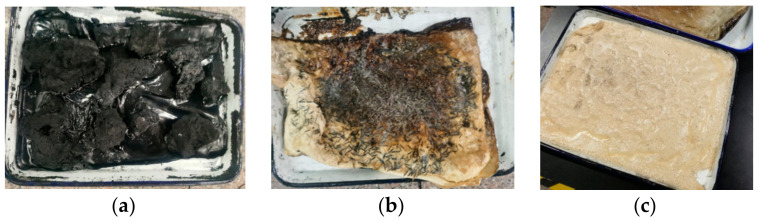
Fiber-reinforced natural latex masterbatches: (**a**) Flocculation of carbon black latex; (**b**) KH560 without latex; (**c**) KH560 with latex.

As shown in Figure 5 subsequently, compounds 5#–7# were mixed with other materials in an internal mixer, vulcanized and shaped, and their performance was tested.

The 1#–7# compounds were kneaded uniformly and then open-smelted by the open mill. The obtained rubber was calendered and oriented along the short-fiber direction. The specific method was as follows:

(a) We adjusted the bar pitch of the open mill to 1 mm, and rolled the roll 5 times along the rolling direction of the open mill. Then, we adjusted the pitch to 3 mm, and paid close attention to passing the roller 5 times along the rolling direction to obtain the short-fiber-reinforced compounds with the most fibers oriented along the rolling direction;
(b) At the end of vulcanization, the performance test should be carried out, and the compounds should be stored for 24 h for standby; this is conducive to the uniformity and stability of the compounds’ properties and the relaxation of the internal stress of the rubber macromolecule.

More specifically, in the 1# compound, the fibers in step (a) were BF9, which were treated again by KH560, and used the formula of all-steel tires.

In the 2# compound, the fibers in the step (a) were BF3, which were only treated with KH550 in the drawing process.

In the 3# compound, the fibers in the step (a) were BF 9, which were only treated with KH550 in the drawing process.

In the 4# compound, the fibers in the step (a) were BF 9, which were treated again by KH560.

In the wet mixing process, the wet mixing method steps were as follows:

In the 5# compound, different from dry mixing, the NR that had been cut into small pieces was replaced by the mixture of NR latex and fibers after drying. (a) No fibers were added in step (a) of dry mixing.

In the 6# compound, different from dry mixing, the NR that had been cut into small pieces was replaced with NR latex and the dried mixture without KH560 fibers in the latex. No fibers were added in step (a) of dry mixing.

In the 7# compound, different from dry mixing, the NR that had been cut into small pieces was replaced by NR latex and the mixture after adding KH560 to the latex. Carbon black was added for drying. No fibers or carbon black were added in step (a) of dry mixing.

#### 2.3.2. Extrusion Experiment of Different Compounds and 3D Physical Model of Extruder Die

**Figure 6:**
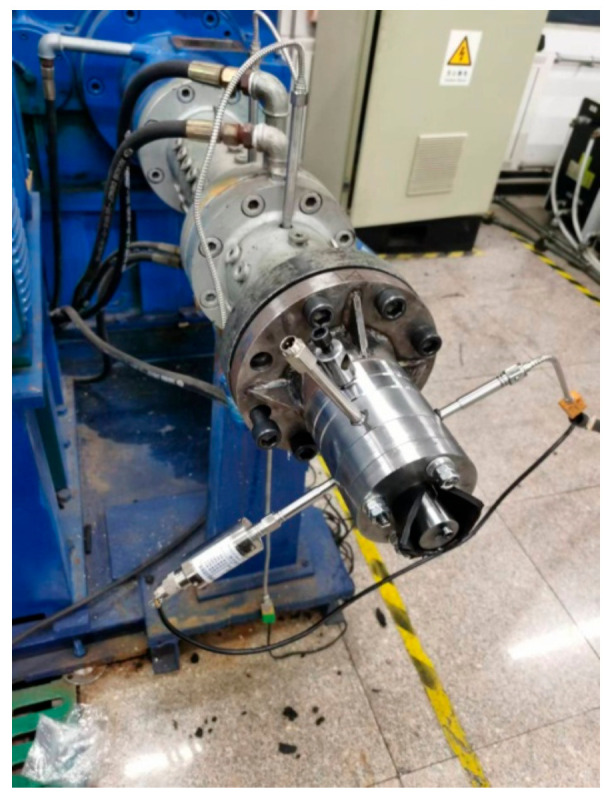
Structure of the die used in the extrusion experiments.

**Figure 7:**
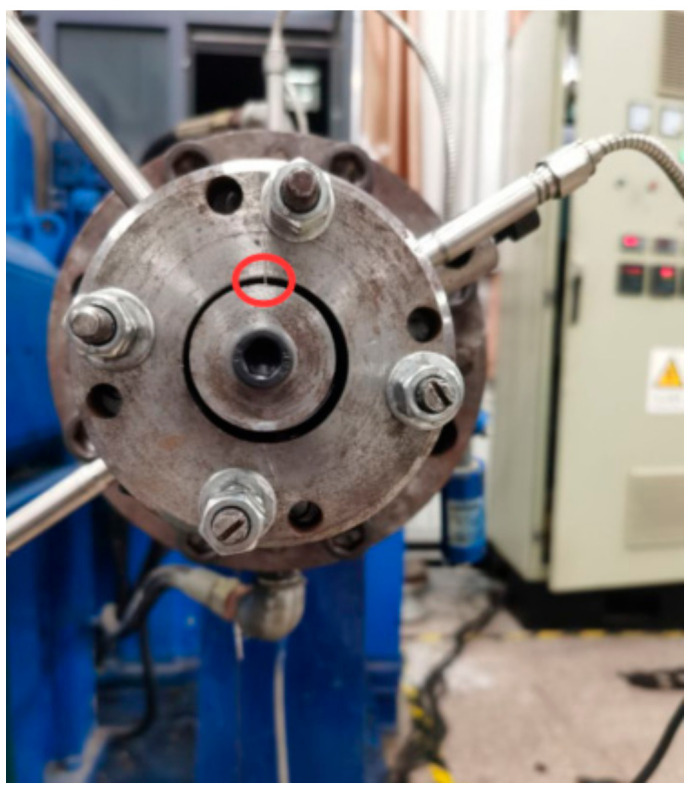
Image of the radial-orientation die.

**Figure 8:**
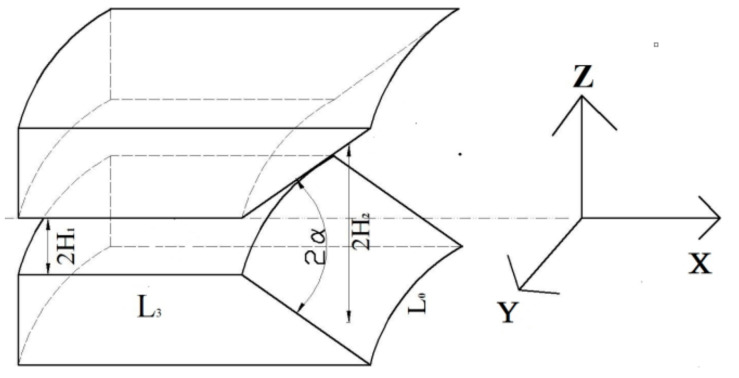
3D physical model of the head.

In this experiment, the split-flow-guided radial-orientation die was used, and the structure of its outlet section is as follows:

Establish the physical model of the orientation of short fibers in the machine head, and cut a section to obtain the 3D physical model that follows. As shown in Figure 6, Figure 7 and Figure 8, this structure is different from the traditional dam structure in that it adopts the annular flow channel, orients the short fibers in the radial direction by the extrusion expansion principle, and then causes the annular shape to become a strip by the joint action of the running speed of the rubber and the cutter fixed at the front of the head. The red circle in the Figure 7 shows the curved runner in Figure 8.

In Figure 8, the *X* axis is the extrusion direction, the *Y* axis is the width direction, and the *Z* axis is the height direction. The radial orientation of the staple fibers is along the *Z*-axis direction and, simultaneously, perpendicular to the *X* axis and the *Y* axis. $H_{1}$, $H_{2}$ is the height of flow channel before and after expansion, α is the expansion half angle, $L_{0}$ is the channel width and the arc length of the outlet part of the circular annular channel, wherein the circular arc portion can be regarded as a straight line, $L_{3}$.

The compounds calendered by the open mill were put into the extruder for extrusion experiments. The experimental conditions were as follows:

The structural parameters of the head: $2H_{2}$ = 8 mm; the length of the middle channel section: $L_{3}$ = 0 mm; the barrel temperature: 80 °C; and the screw speed: 35 r/min. The vulcanization test was carried out after the extruded rubber was cooled for 8 h.

NR/BF reinforced composites were prepared by mixing 3 phr BF with natural rubber, then mixing in an internal mixer, vulcanizing and forming. After parking, the effects of the length of BFs on the mechanical properties and dynamic mechanical properties of NR/BF-reinforced composites were studied and compared with the wet mixing method.

### 2.4. Characterization

#### 2.4.1. Physical and Mechanical Property Test and Wear Resistance

The physical and mechanical properties tested mainly included 100% constant elongation stress, tensile strength, tear strength, etc. The tests were carried out on the UT-2060 tensile testing machine. The tensile speed was 500 mm/min, and the test temperature was room temperature. We recorded the tensile strength, 100% constant elongation stress and elongation at break.

The DIN abrasion test of the vulcanization was carried out according to the latest relevant national standards, and the DIN abrasion stroke was 40 cm. The test process was conducted as follows:

##### Preparation of the Test Sample

**Figure 9:**
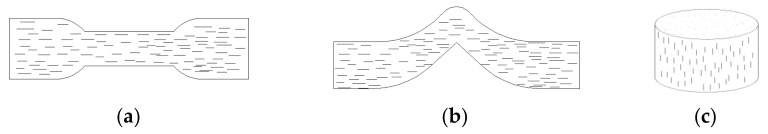
Samples for test. (**a**) Sample for tensile; (**b**) Sample for tear; (**c**) Sample for DIN wear.

Because of the radial orientation of the short fibers, the vulcanizate has anisotropic properties. Therefore, in order to characterize the degree of anisotropy of the compound and to compare and analyze the influence of the properties of the vulcanizate with short-fiber orientation, performance test specimens were prepared on GT-7016-AB pneumatic automatic slicer along different orientation directions of the short fibers, as shown in Figure 9. The short horizontal lines in Figure 8 represent the direction of the short fibers and their orientation, where the test of sample (a)~(c) is recorded as either the S test [1] or V’ test. In this experiment, both S and V’ tests are along the fiber arrangement direction, but one is the extrusion direction of the fibers and the other is the radial direction of the fibers.

**Figure 10:**
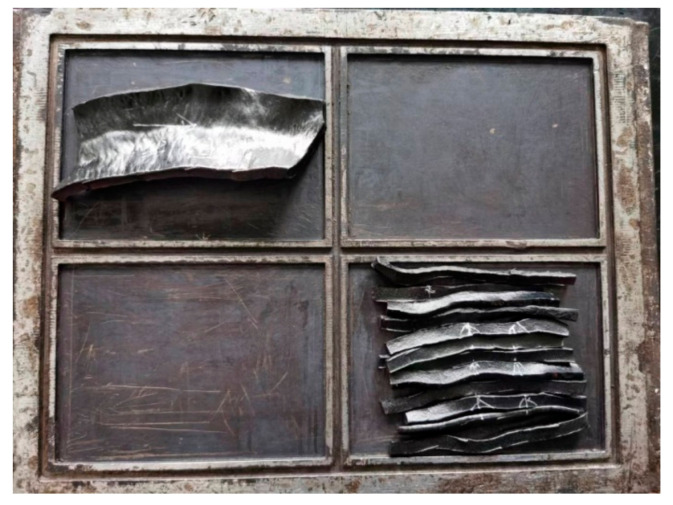
Samples for S and V′ tests in the mold.

As shown in Figure 10, the process of the V’ test was as follows: we cut the compounds into strips along the vertical direction of the axial direction. The thickness in the axial direction was 2 mm, totaling 24 g. We placed the compounds along the mold direction on the bottom right, shown in Figure 10, which was the radial direction. This method can determine the comprehensive physical and mechanical properties of the compounds in the radial direction after extrusion. In this paper, only the physical and mechanical properties of the compounds in the radial direction—V′ direction—were reflected.

##### Performance Test

The physical and mechanical properties were tested on the UT-2060 tensile testing machine and GT-2012-D wear testing machine, in strict accordance with the test operation procedures and specifications, and the data were recorded completely.

#### 2.4.2. SEM Scanning Test Process

The JSM-7500F scanning electron microscope produced by Japan Electronics Company was used for fiber SEM to observe the morphology of the sample. The sample was brittle broken with liquid nitrogen, and the section was sprayed with gold. The test acceleration voltage was 5 kV.

#### 2.4.3. Payne Effect

The RPA2000 rubber processing analyzer was used to conduct temperature change treatment on the mixed rubber first. The setting time was 0 min → 2.5 min → 5 min, and the corresponding temperature was 60 °C → 160 °C → 60 °C. Then, the temperature was adjusted after a delay of 5 min, and then the strain scanning was performed.

#### 2.4.4. Mooney Test Process

The UM-2050 test instrument was used to conduct Mooney test.

#### 2.4.5. Dynamic Thermomechanical Analysis

The dynamic thermal mechanical analyzer (DMA) can obtain the dynamic storage modulus, loss modulus and loss angle tangent of the material. The temperature range was 65−65 °C.

#### 2.4.6. Hardness

The LX-A hardness tester produced by Shanghai Liuling Instrument Factory was used to test the hardness of the vulcanizate.

#### 2.4.7. Three-Dimensional Topography Test

The surface morphology of the metal was observed by the LEXT OLS5000 3D laser measuring microscope, and the amount of wear was obtained by measuring the volume reduction of the metal grinding head.

#### 2.4.8. Carbon Black Dispersion Meter Test

The cut surface of the vulcanizate was irradiated with light at a certain angle. If the carbon black was well-dispersed in the vulcanizate, the cut vulcanizate surface would have a flat and less defective structure; the dispersion degree of carbon black in vulcanizate could be characterized according to the size and frequency of these bumps. These conditions on the vulcanized rubber surface were directly reflected on the screen and recorded as digital images. The digital image was processed by a computer, and the dispersion grade of carbon black was automatically evaluated according to ISO 11345-2006. The test threshold was set to “auto”, and the standard was “ISO11345CB” for 10 levels of judgment. Each sample was tested five times, and the average value was taken as the judgment level.

## 3. Analysis and Discussion of Test Results

### 3.1. Vulcanizate in Filler System, Vulcanization Performance, Physical and Mechanical Properties and Dispersion Effect, SEM Analysis

#### 3.1.1. Vulcanization Performance Analysis 

The following is the comparison of vulcanization properties of various compounds before extrusion:

It can be seen from the Table 2 and Table 3 that the *M_L_* value and *M_H_* value of BF9 and BF3 are both decreased relative to those in the wet mixing, which indicates that after the addition of fibers, the fluidity of the compounds after dry mixing is better. When the fibers are wet-mixed, the fibers and the latex are evenly mixed. It can be seen from the increase in stiffness of the composites that the *M_H_-M_L_* value is also increased. Compared with the formulation of all-steel radial tires (compound 1#), the cross-linking density of the BF9-reinforced composite is slightly lower than that of dry NS, but the difference is small. To a certain extent, the increase in the *M_H_-M_L_* value contributes to the directional arrangement and stretching crystallization of the molecular chain, and the tensile strength and hardness are increased. This can be seen from the physical and mechanical properties of the composites in Table 3.

However, if the cross-linking density *M_H_-M_L_* value is excessively increased, the cross-linking network hinders the directional arrangement and crystallization of the molecular chain and, at the same time, the uneven distribution of the cross-linking bond is aggravated, resulting in more uneven stress distribution, and the tensile strength decreases.

At the same time, the Mooney viscosity of rubber composites was also increased by wet mixing. This was because BFs were added into the latex during wet mixing and evenly dispersed, which increased the overall Mooney viscosity of the compounds. Although the macromolecular chain of rubber molecules in the mixing process should be more strongly affected by the shear flow field of the rotor of the internal mixer from the mixing mechanism after two instances of mixing, the overall stiffness of the composites is improved due to the dispersion of BFs, and the Mooney, *M_L_* and *M_H_* values are higher. With an increase in KH560, the KH560 Si–O bonding layer is uniformly formed on the surface of BFs, which expands the contact area between BFs and the rubber matrix, limits the deformation of the rubber matrix, and improves the interface interaction. Macroscopically, the cross-linking density of the composites after adding a coupling agent in wet mixing is slightly lower than that of the dry method.

Compared with those before extrusion, the *M_H_-M_L_* values of most of the compounds are also decreased; this is because after entering the extruder head, the compounds were extruded by the screw, and some macromolecular chains were broken, and the flexibility was increased. The positive curing time T90 decreased correspondingly, which was due to the strong shear action of the extruder screw, which generates a large amount of heat in the compounds and forms viscous dissipation, thus shortening the curing time of the compounds.

It can be seen from Table 1 and Table 2 that the Mooney viscosity of the compounds after extrusion is greatly reduced, which is due to the heat generated by the shear flow field and the tensile flow field of the screw shape and the internal configuration of the die, which increases the flexibility of the macromolecular chain, reduces the viscosity of the compounds, enhances the fluidity and greatly increases the processing performance. The Mooney viscosity of the wet-mixed compounds is greater than that of the internal mixer. This is because during the wet mixing, the fibers were mixed with the latex first and evenly dispersed therein. The fibers have better rigidity and elastic modulus and can be better dispersed in the latex matrix than those in the internal mixer. It can be seen from the RPA test results that the dispersion of filler in the wet-mixed compounds is better.

It can be seen from the table that the Mooney viscosity of the compounds with the all-steel radial tread formula is similar to that of the compounds with BFs, and their processability is not different, but it is different from that of the compounds in wet mixing.

By impregnating BFs with the latex matrix, the latex matrix can be fully contacted with BFs, and a natural rubber film closely bonded with BFs can be formed on the surface of BFs, which can better bond with the rubber matrix during the mixing process.

#### 3.1.2. Physical and Mechanical Properties

The physical and mechanical properties of vulcanized samples are shown in Table 4 and Table 5.

In Table 5: the meaning of the parameters σ_1_ and σ_2_ is the same as that in Table 4.

It can be seen from Table 4 and Table 5 that the hardness of wet mixing after extrusion is somewhat low and the dispersion of carbon black is high. The Mooney viscosity is better than when not extruded. The tensile strength and DIN abrasion of the wet-mixed compounds after extrusion (with N234 in latex, 7#) are increased by 1.79 MPa and 13.3%, respectively, compared with the traditionally mixed compounds (4#), indicating that the wet-mixed compounds have good mechanical and physical properties.

Compared with the performance test data of the mixed compounds prepared by the traditional method, it can be seen that the comprehensive performance of the mixture prepared by this method is far better than that of the mixture prepared by the traditional dry method. BF has better compatibility in the rubber latex matrix and eliminates the interface effect in the bonding process. Moreover, BF can improve the dispersion of reinforcing filler in the rubber matrix. After the latex and carbon black are mixed, the latex can be flocculated by means of a high-speed shear stirring device.

It can be seen from Table 4 and Table 5 that the hardness, 100% constant tensile stress, and tear strength of the compound increase and the elongation at break decreases with an increase in BF length.

This is mainly because BFs are dispersed in the rubber to play the role of skeleton reinforcement, which improves the rigidity and hardness of the rubber. With a decrease in the length of basalt short fibers, the number of overlapping points between basalt short fibers increases, forming an interlaced network structure in the rubber matrix and hindering the sliding of the surrounding rubber polymer chain. Meanwhile, the binding force between the fibers and the rubber matrix will increase the tensile stress and tear strength of the rubber sample, and the elongation at break will decrease.

However, with an excessive increase in the short-fiber lengths, two stress concentration points will be generated in the compounds at both ends of the basalt short fibers when the sample is stressed. The stress concentration points will increase and the possibility of damage will also increase, which will reduce the tensile strength. The DIN wear amount shows an increased trend. This may be because when the BFs were added in a small amount and had short lengths, the short fibers could limit the deformation ability of the rubber matrix when it was rubbed under the orientation state. To a certain extent, the development of cracks is limited. At the same time, when the compounds are worn off, the short fibers are exposed on the surface to wear the short fibers, which reduces the wear amounts to a certain extent.

The fibers treated with silane coupling agent KH560 used in this experiment have good compatibility with latex. The strong shear and high-speed stirring have a wall-breaking effect on the latex. The strong internal friction between BFs and the outer protein wall of the latex leads to a wall-breaking effect. After wall breaking, the macromolecular chains of the core part of the latex will be wound on the fiber surface and cross-linked after vulcanization. The physical and mechanical properties of the extruded material are higher in the radial direction. The interface between reinforcing fibers and the rubber matrix can improve its dispersion in rubber matrix and realize acid-free flocculation of natural latex.

From the three semi-finished short-fiber-reinforced latex products dried in Figure 5, it can be seen that after adding KH560 to the solution, the cohesion of its fibers decreases, and its dispersion is better than that of the fibers directly treated with KH560. This is because KH560 is uniformly dispersed in the latex solution, and the basalt-fiber dimension and the macromolecular chain of the core part of the latex after wall breaking can be more bonded together through Si–O bonds, which shows that its physical and mechanical properties are better and that it has higher hardness and lower rolling resistance.

It can be seen from Table 3 that the hardness, 100% constant tensile stress and tear strength of the rubber sample are increased and the elongation at break is decreased with the addition of basalt short fibers compared with the rubber without basalt fibers. This is mainly because basalt short fibers are dispersed in the compounds to play the role of skeleton reinforcement, which improves both the rigidity and the hardness of the compounds.

When the length of basalt fibers is relatively short, an increase in the length of basalt short fibers causes the whole fibers to be more closely combined with rubber in the rubber matrix. Compared with 3 mm BF, one fiber in 9 mm BF has more adhesive, forming an interlaced network structure in the rubber matrix and hindering the sliding of the surrounding rubber high-resolution chain. Meanwhile, the binding force between the fibers and the rubber matrix will increase the tensile stress and tear strength of the sample under stress, and the elongation at break decreases.

The tensile strength tends to increase first, mainly because when the short fibers are added in a small amount, the short fibers can limit the deformation of the rubber matrix to a certain extent under external force. The external force first acts on the interface phase between the short fibers and the rubber matrix, and then transfers to the rubber matrix, so the tensile strength increases.

**Figure 11:**
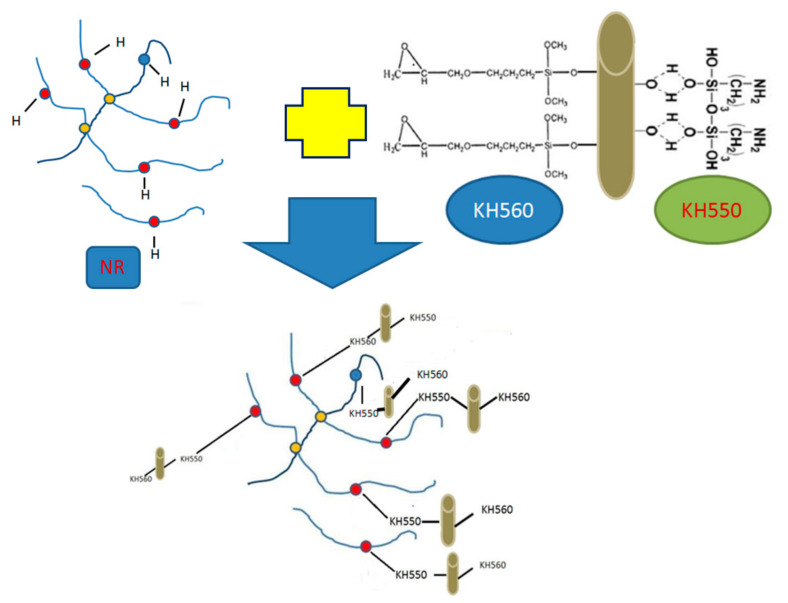
Coupling mechanism of KH550/KH560 with BFs.

Microscopically, as shown in Figure 11, when the deformation of the sample cannot be alleviated by the movement of its own chain segment, most of the stress will be transferred to the short fibers in the rubber matrix, and since the BFs and the rubber matrix form a good interface phase through KH550 and KH560, the stress can be smoothly transferred to the rubber matrix through the interface phase; the BFs restrict the movement of the rubber molecular chain around it, and more energy is needed to destroy the BFs or pull the BFs out of the rubber matrix. As a result, the 100% constant tensile stress, tensile strength and hardness of the sample are increased, and the elongation at break of the rubber sample is decreased. At the same time, the addition of KH560 is beneficial in improving the lubrication and flexibility of the rubber molecular chain and improving the fluidity of the compounds. This is because the hydroxyl end -OH of BFs reacts with Si–OH in silane coupling agents KH550/KH560, and the -CH_3_O bond reacts with the -OH bond to form CH_3_OH, dehydration forms a Si–O bond, and Si in the hydrolyzed coupling agent is connected with C atoms in the compounds to form a fiber–rubber ternary combination, which improves the compatibility of the fiber and rubber matrix. The double-bond C atom on the main chain of NR molecule can undergo an addition reaction, while other C atoms are α type. The C atom is easily dehydrogenated and connected to other monomers, so the monomer can be connected to any C atom on the NR main chain.

#### 3.1.3. SEM Analysis

**Figure 12:**
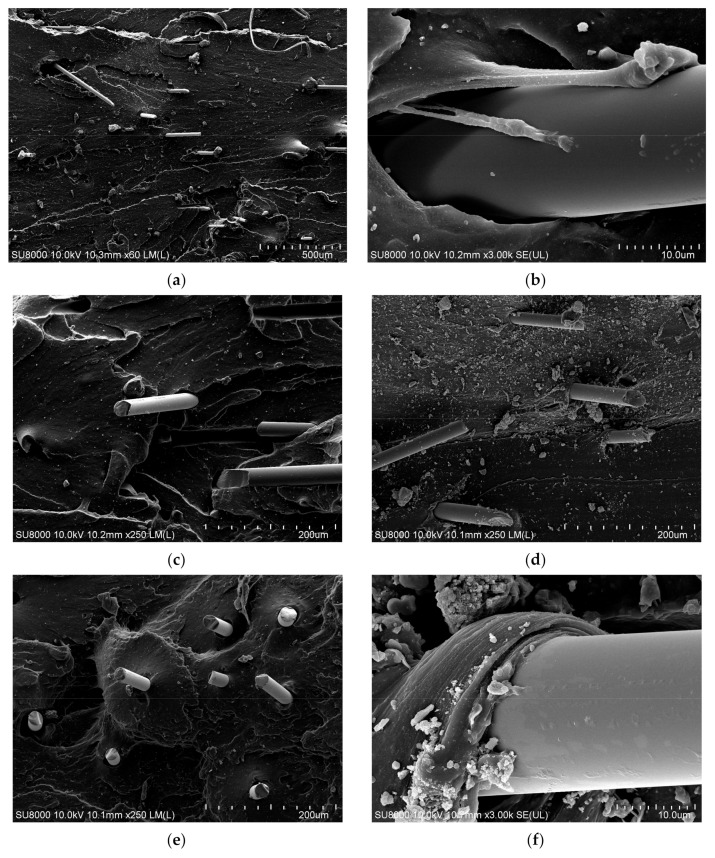

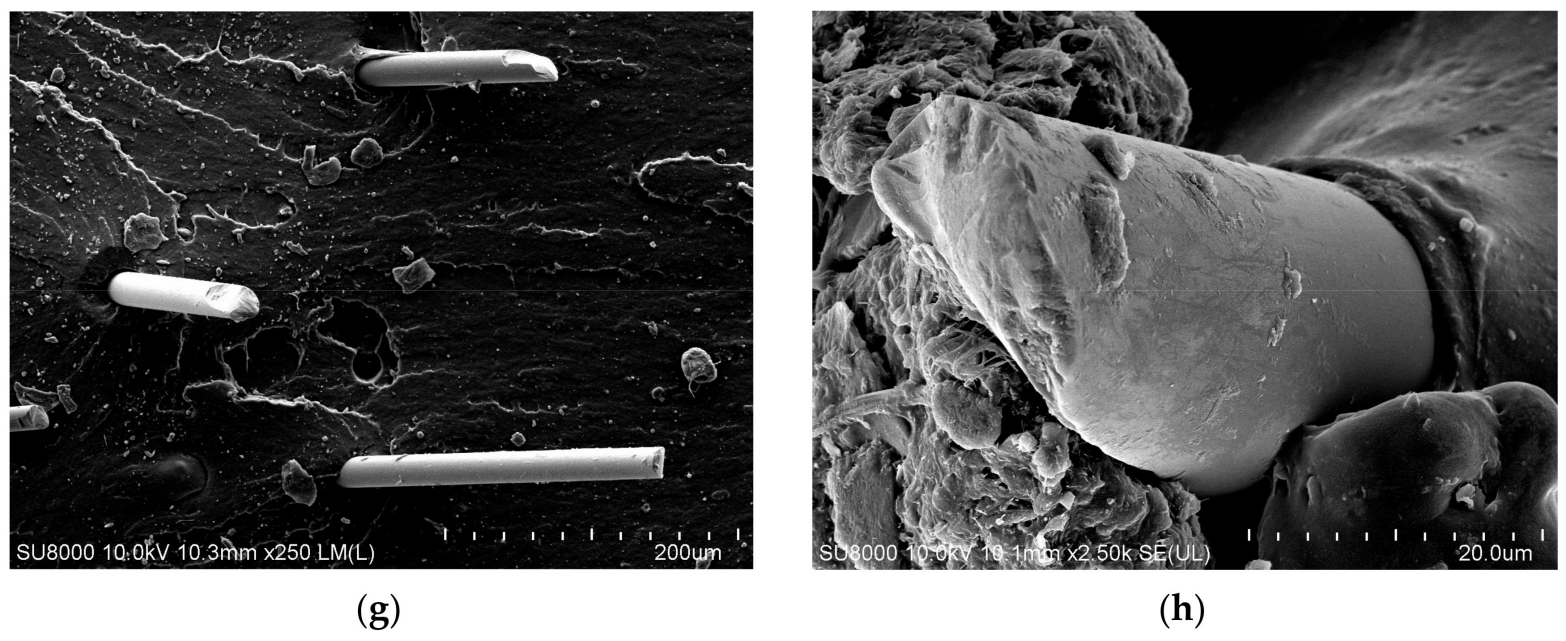
Electron micrographs of adhesion between BF9 and rubber matrix under different treatment conditions: (**a**–**c**,**e**) are the images of fiber-reinforced composite without KH560 treatment, (**d**,**f**–**h**) are the images of fiber-reinforced composite without KH560 treatment.

Figure 12a–c,e contains images of fiber-reinforced composite without KH560 treatment. The cross section of the 9 mm tensile sample shows that the adhesive effect is good, and the fibers in the rubber matrix will drive the rubber. Figure 9a shows the fiber-reinforced composite without KH560 treatment and only with KH550 treatment in the drawing process. From the enlarged Figure 12b, it can be seen that the adhesive is attached to the fiber pull-out interface, which proves that the adhesion between the fiber and the adhesive is good.

However, in Figure 12d,f,h, more adhesive is obviously attached after KH560 treatment, which indicates that the fiber has strong adhesion during the tensile process of the sample after KH560 treatment and can make the adhesive adhere to the fibers. It is preliminarily proven that the fiber has better adhesion after being treated with coupling agent KH560 again.

Figure 12 displays electron micrographs of samples of 9 mm BF-reinforced rubber product after dry mixing. For the cross section of the tensile sample treated with KH560, it can be seen from Figure 12 that after the coupling agent is added, the fiber surface is rough, and the adhesion between the rubber and the fibers is increased to a certain extent, which is manifested in its high tensile strength. However, the adhesiveness of the fibers was greatly improved after the addition of KH550 by fiber drawing. After the addition of KH560, the adhesiveness of the fibers was correspondingly improved, but the improvement was not too large. The adhesiveness of the original KH550 adhesive caused most of the fibers to cover the fiber surface. After the addition of KH560, the effect was improved, but the enhancement was not strong due to the previous effect of KH550, so the improvement was not too large. However, compared with the fibers without KH550, there is obvious adhesion between the fibers and the rubber matrix, which proves that the fibers have better adhesion after adding KH560. In principle, this is due to the joint effect of KH560 and KH550 on their surfaces.

The electron micrographs are shown in Figure 12. It can be clearly seen from Figure 12 that the BFs at the tensile section are not completely pulled out, and the end is in a state of being pulled off, which indicates that the short fiber effectively withstands the tensile stress during stretching, and serves as a stress concentration point in the rubber matrix to enhance the rubber strength.

At the same time, there is an obvious glue-hanging phenomenon on the surface of the short fiber, which indicates that after the short fiber is modified by KH560, the interface layer of BF–KH560–natural rubber + BF–KH550–natural rubber is formed, which improves the adhesion between BFs and the rubber matrix, which corresponds to the experimental results and analysis of mechanical properties of the composite. Figure 12g shows the appearance and structural characteristics of the void after the short fiber is pulled out. It can be seen from the figure that the surface of the gap is uneven after the short fiber is pulled out. It shows that a large amount of rubber matrix is attached to the surface of the pulled short fiber, and the side shows that the short fiber and the rubber matrix form a good interface phase, which improves the adhesion between the short fiber and the rubber matrix.

#### 3.1.4. Three-Dimensional Topography Test

**Figure 13:**
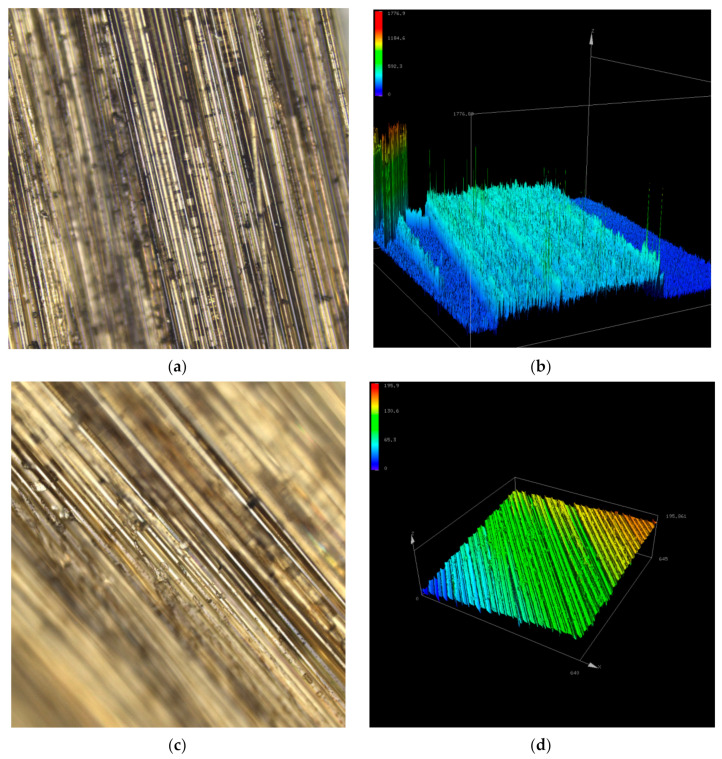
Three-dimensional micrographs of BF9. (**a**) 20-fold enlarged fiber image of BF9 treated with KH560; (**b**) The contour map of BF9 treated with KH560 which is enlarged by 20 times; (**c**) 20-fold enlarged fiber image of BF9 treated without KH560; (**d**) The contour map of BF9 treated without KH560 which is enlarged by 20 times.

It can be seen from Figure 13 that the three-dimensional morphology observation electron microscope image of BFs at 20 times magnification shows that the fibers treated with KH550 and KH560 have better surface roughness, and many particles of KH560 coupling agent are attached to the fibers treated with KH550 and KH560 compared with the fibers treated with only KH550.

It can be seen from the height contour map in Figure 13b,d that the height difference of the surface undulation of the fibers treated with KH560 is relatively dense and the undulation is relatively large, so the fiber profile cannot be clearly seen. This is because the fiber surface roughness is increased as a result of the pretreatment of the fiber surface with KH560 and KH550, so there are more undulating height lines. This also proves that the adhesiveness of the fiber after adding KH560 on the basis of KH550 pretreatment is better than adding KH550 only in the drawing process of basalt fibers. The picture in Figure 13 is blurry because the surface of the basalt fiber is rough, so it can not reach a very clear degree.

#### 3.1.5. BF on Dynamic Viscoelasticity of Composites

**Figure 14:**
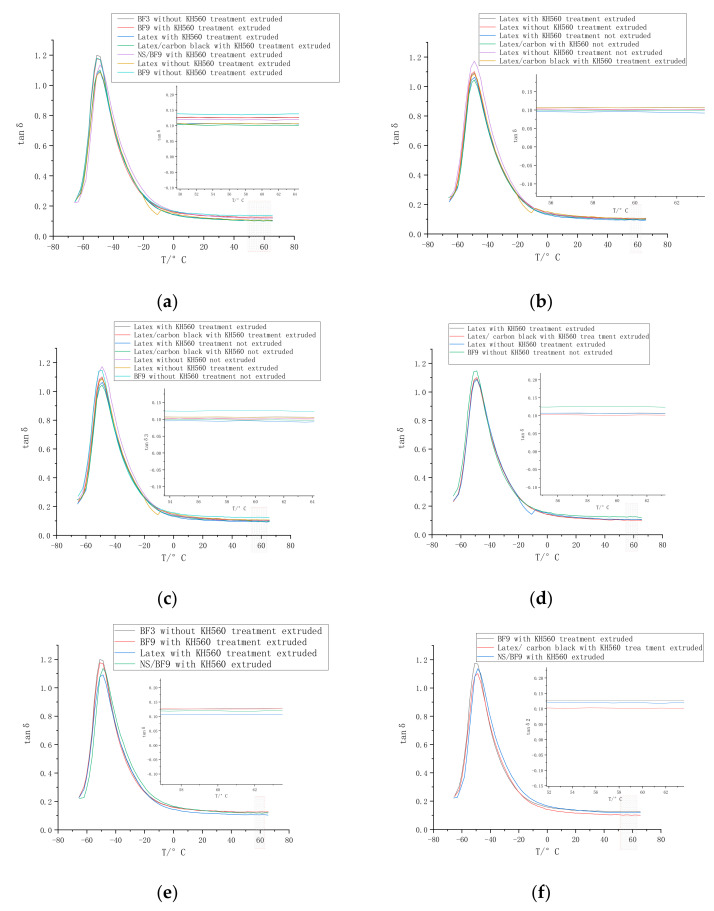
Results of DMA tests. (**a**–**f**): tanδ–T Curves of the composites.

As can be seen from Figure 14, the curves of the loss factors with the temperature changes in the composites. When latex is added, the rolling resistance of the composites at 60 °C is significantly reduced, and the wet-sliding resistance at 0 °C is not much different. The rolling resistance at 60 °C of 9 mm BF is smaller than that of 3 mm BF.

It can be seen from the figure that the rolling resistance of the wet-mixed rubber is reduced correspondingly and the wet skid resistance is slightly reduced.

The dynamic viscoelasticity of vulcanized rubber includes rolling resistance, wear resistance and wet-sliding resistance. The rolling resistance reflects the fuel economy, the wear resistance reflects the durability and service life of the tire, and the anti-skid performance is directly related to the safety of the tire. The curve of loss factor with temperature in NR/3 mm BF composite basically overlaps with the curve of NR/9 mm BF composite in Figure 14, but the loss factor of NR/3 mm BF is slightly larger than that of NR/9 mm BF, meaning that and NR/9 mm BF is slightly better. This means that the composites prepared by these two methods have little difference in moisture resistance and rolling resistance. At 0 ℃, the 3 mm BF and 9 mm BF are 0.162764 and 0.159801, respectively, and at 60 ℃, the 3 mm BF and 9 mm BF are 0.126529 and 0.125668, respectively.

It can be seen from t Figure 14 that the composites prepared by NR/9 mm BF/DZ composite and NR/9 mm BF/NS composite have little difference in moisture resistance and rolling resistance. Therefore, the use of the DZ delay formula can better replace NR/9 mm BF/NS composites, and can delay the curing process of the composite and prevent scorching of the composites. However, the rolling resistance of the composites prepared by wet mixing method is obviously improved compared with the traditional mixing method; at 0 ℃, 9 mm BF–latex- carbon black and 9 mm BF are 0.142294 and 0.159801, respectively; at 60 ℃, 9 mm BF–latex- carbon black and 9 mm BF are 0.0999589 and 0.125668, respectively, with little difference in wet-sliding resistance and large difference in rolling resistance.

The interface interaction between the wet-mixed basalt fiber and the rubber matrix becomes stronger, and the movement of the rubber molecular chain is restricted, which increases the elastic modulus of the composite material. Moreover, BF has been fully dispersed in the latex matrix before entering the internal mixer. Compared with the internal mixer, the dispersion of the wet-mixed basalt fiber is relatively more uniform. At the same time, BF becomes the stress point. Because most of the fibers are oriented in the radial direction after the composites pass through the short-fiber-orientation machine head, their wet-sliding resistance in this direction is significantly improved. However, the rolling resistance as a whole is slightly increased due to a decrease in the cross-linking density of the rubber, but it is almost the same as that when it is not extruded. This is because the special rigidity of the short fibers causes the fibers to be oriented in the radial direction. With the addition of KH560, on the basis of the original KH550, the surface of BFs uniformly generates a KH560 Si–O binding layer, which expands the contact area between BFs and rubber matrix, limits the deformation of the rubber matrix and improves the interface interaction; furthermore, its rolling resistance is relatively reduced. The loss factor at 0 ℃ and 60 ℃ is smaller than that of dry mixing.

The reason why the addition of short fibers reduces the rolling resistance of the tire is that in the radial direction, the addition of short fibers can improve the rigidity of the tire, so that the sinking amount of the tire under the same load during driving is significantly reduced; that is, the deformation of the tire is reduced, and the rolling resistance is also reduced. Moreover, the friction coefficient of the tread can be reduced and the rolling resistance at 60 ℃ is reduced accordingly.

When the external force is applied, the rubber matrix is transferred to the fibers through the interface phase.The loss factor is denoted tan δ. It indirectly reflects the interface action between the rubber matrix and BFs. In general, for tan δ, the larger the peak value, the worse the interface between basalt short fibers and the rubber.

In wet mixing, the glass transition temperature corresponding to the peak value of tan δ is also decreased, which may be due to the low glass transition temperature of wet mixing. When the glass transition temperature of rubber is reached, the basalt short fibers can also play a reinforcing role in the rubber matrix. Compared with dry mixing, it does not cause brittle failure of rubber at low temperatures. The addition of basalt short fibers reduces the proportion of rubber in the composites; usually, the maximum value of tan δ will decrease with an increase in filler amount.

The smaller the value of tan δ, the stronger the interaction between BFs and the rubber, the more restricted the activity of rubber molecular chain segment and the higher the storage modulus G′, resulting in reduced loss of vulcanizate. The peak value of tan δ decreases. Figure 6 shows the wet-sliding resistance and rolling resistance of the composite sample. It can be seen that the addition of short fibers in the orientation direction can improve the rolling resistance of the tread rubber.

Tan δ at 60 °C is shown in Figure 14; that is, the rolling resistance of the composites. In the range of 40–60 °C, the loss factor value of composites with BFs is higher than that without BF. The rolling resistance of extruded material is slightly higher than that of non-extruded, and the wet slip resistance of extruded rubber is better. However, this does not indicate that the degree of short-fiber radial orientation in non-extruded rubber is higher. The comparison of rolling resistance is that under the same conditions, that is, under the same extrusion conditions, the lower the rolling resistance in the radial direction, and the higher the tensile property in the orientation direction, the better the radial property, and the higher the orientation degree. This can be seen from the fact that the tensile property of the composites before extrusion is higher than that after extrusion, but the rolling resistance is lower. After the extrusion and shearing of the extruder screw, a large amount of heat is generated in the rubber, and the viscosity of the composites decreases. The analysis shows that the composite material begins to soften at a high temperature, and the macromolecular chain in the rubber matrix is interrupted by the shearing of the screw. The composites were extruded with a high viscosity and poor fluidity. After extrusion, the overall cross-linking density of the composites decreases and the viscosity decreases due to the shearing heat of the screw; the specimen is more likely to be deformed by external force. The restriction effect of the cross-linked network formed by basalt fibers and the rubber matrix on the rubber macromolecular chain is weakened, and the friction between basalt fibers, filler and the rubber matrix in the composite increases the energy loss, so the rolling resistance of the composite is also improved.

#### 3.1.6. BF on Payne Effect of Composites

**Figure 15:**
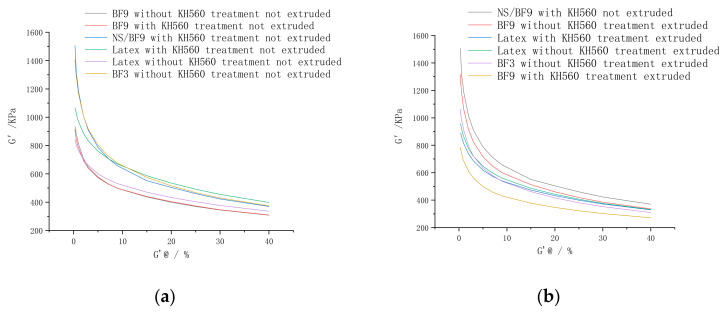

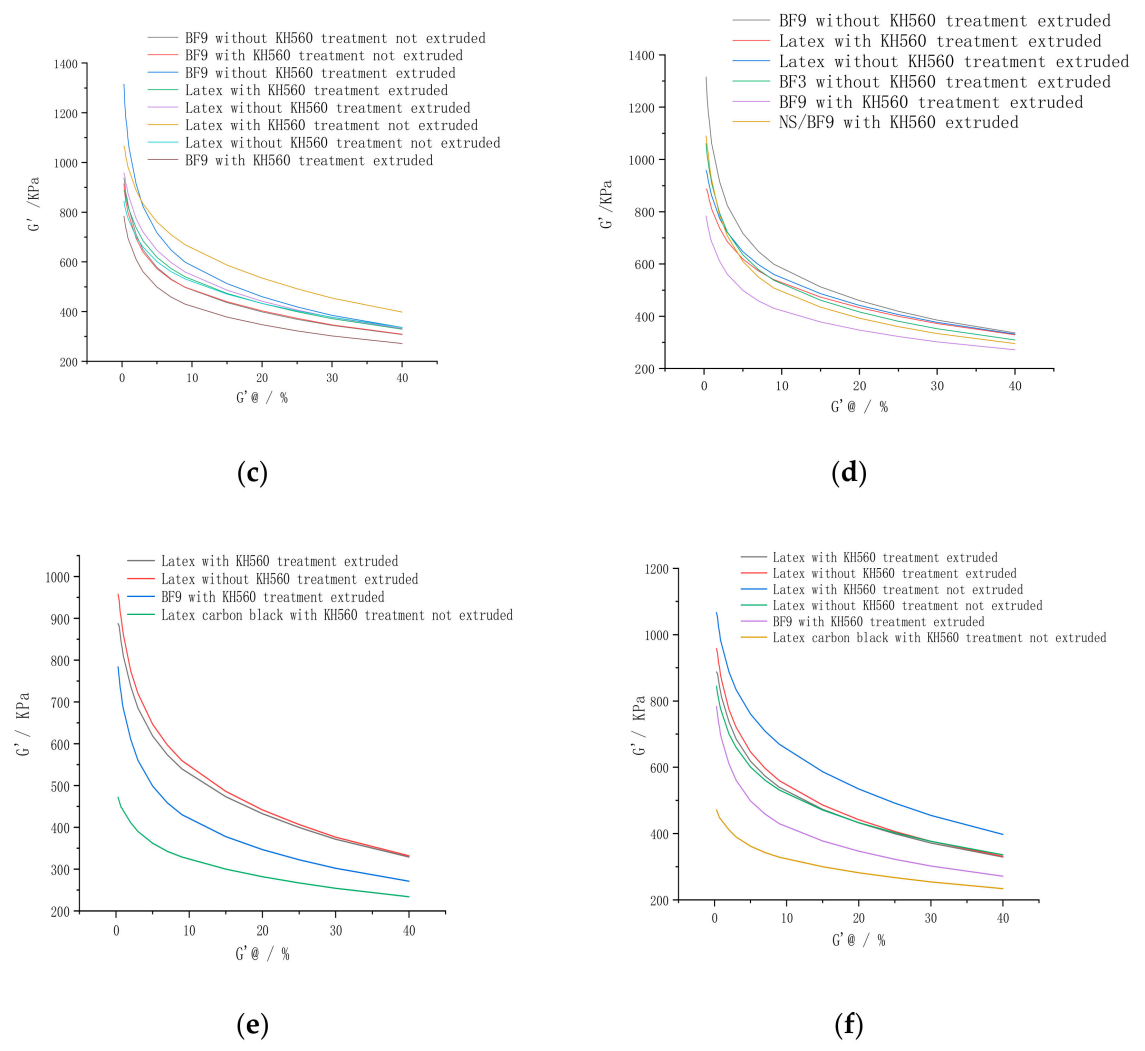
Results of RPA tests. (**a**–**f**): Viscoelasticity Curves of the composites.

It can be seen from Figure 15 that the dispersion in the filler of the extruded rubber is better than that in the non-extruded rubber. Under the same extrusion conditions, the dispersion effect of wet mixing is the best. It can be seen from Figure 15 that the ΔG′ of the NR/BF-reinforced composites mixed by wet mixing is significantly reduced, which indicates that the BFs mixed by wet mixing can limit the deformation of the rubber matrix to improve the interfacial adhesion between the BFs and the rubber matrix, and the BFs can be better dispersed in the rubber matrix.

The value of ΔG′ in the NR/BF composites treated with KH560 is relatively low, as seen in Figure 15. KH560 strengthens the adhesion between BFs and the rubber matrix. When treated with 1.5 g/L KH560 solution, the dispersion of BFs in rubber matrix is better than that of untreated NR/BF composites. This is because the secondary treatment of KH560 on the basis of the original KH550 treatment increases the adhesion of chemical bonds between the fibers and the rubber matrix, and the fibers are not easy to agglomerate during mixing, so their dispersion is increased. In addition, as shown in Figure 15, when carbon black was added during wet mixing, the dispersion of short fibers was the best when the short fibers and rubber were blended. This is because the carbon black is uniformly mixed with the latex before mixing, and the carbon black is uniformly dispersed in the latex matrix.

It can be seen from Table 4 and Table 5 that after extrusion, the physical properties are better, which indicates that the dispersion of BFs in the rubber matrix and the adhesion between BFs and the rubber matrix are better. This is because in wet mixing, before entering the internal mixer, the fibers have been fully dispersed in the latex solution, and compared with mechanical force, the fibers and natural latex molecular chain are more easily combined; the agglomeration of BFs is the weakest, so the dispersion of filler is the highest. Moreover, the dispersion of natural latex treated with KH550 and KH560 is better than that of natural latex without KH560 treatment and only treated with KH550.

After extrusion, the value of ΔG′ decreases, indicating that with the addition of KH560 treatment, the agglomeration of BFs weakens, and the dispersion of BFs in the rubber matrix improves. This is mainly due to the effect of the shear flow field and the tensile flow field of the orientation die, which makes the fibers in the rubber material arranged regularly in all directions, and the fibers are fully dispersed and mixed for the first time under the action of the extruder screw, so that the dispersion of the filler in the rubber material increases correspondingly. In addition, the dispersity of 3 mm reinforced basalt fiber is lower than that of 9 mm reinforced rubber composite. This may be because the stress of the fiber is shorter than that of the rubber composites, and the agglomeration of 3 mm BF becomes the cross-linking concentration point, resulting in a large number of uneven cross-linking networks. As a result, the rubber matrix cannot transfer the stress well during the stress action, and becomes the stress concentration point. Therefore, the dispersion and physical and mechanical properties of the filler are reduced.

## 4. Conclusions

(1) The physical and mechanical properties of wet-mixed basalt fiber-reinforced rubber products under the same conditions are better than that of dry-mixed basalt fiber-reinforced rubber products. The dispersion of fibers in the rubber matrix is the best and the rolling resistance is the lowest. After adding KH560, compared with not adding KH560, the properties, dispersion, carbon black dispersion and wet-sliding resistance are improved to a certain extent. The BFs added with KH560 by wet mixing can limit the deformation of the rubber matrix to improve the interfacial adhesion between BFs and the rubber matrix, and BFs can be better dispersed in the rubber matrix, compared with that before extrusion, except that the rolling resistance of the rubber was slightly increased due to the shear heat generation of the extrusion screw; the other properties were significantly increased. By adding DZ and CTP into the curing system, the curing process of the rubber compound was delayed, and the scorch resistance of the short-fiber-reinforced rubber products was enhanced.

(2) After the compounds were extruded by the short-fiber orientation die, the fluidity increases and the positive curing time decreases. The basalt-fiber latex was added in the wet mixing. The fiber dispersion is the best, and the Mooney viscosity of the compounds is increased. Most of the fibers are oriented in the radial direction, so the rolling resistance performance in the radial direction is relatively increased, and the wet-sliding resistance is relatively decreased compared with the dry method, but the range is not large. The properties of the composites, such as carbon black dispersion, filler dispersion, rolling resistance and wet-sliding resistance, are the best after the latex, carbon black and fibers were premixed and then mixed by a mixer.

(3) In this paper, the qualitative characterization experiments on the orientation direction of the vulcanizate was carried out. Through the qualitative characterization experiment of segmented cutting and vulcanization in the radial direction of the rubber, it is shown that the short-fiber orientation head can greatly improve the radial orientation of the short fibers in the radial direction. The better the radial performance, the better the dispersion of carbon black and filler, indicating that the head has a better radial orientation effect. Moreover, the adhesiveness of basalt fibers is greatly improved after adding KH550 by fiber drawing, and the adhesiveness of basalt fibers is correspondingly improved after adding KH560, which can realize the high-value application of basalt fibers in the formulation system of rubber composites.

## Figures and Tables

**Table 1:** The mass fraction of each component in the delayed formula and all-steel tire formula in the experiment.

Delayed Formula	Formulation (phr)	All-Steel Tire Formula	Formulation (phr)
NR	85	NR	100
BR	15	Plasticizer	0.08
Carbon black N234	43	Carbon black N234	45
ZnO	4	ZnO	3.5
SAD	2	SAD	2
4020	2	4020	1.5
Microcrystalline wax	1	Microcrystalline wax	1
S	1	S	1
DZ	1.8	NS	1.6
CTP	0.3	RD	1.5
RD	1	Si69	1
Si69SiO_2_	0.55	SiO_2_	8

**Table 2:** Results data of experiments for vulcanizate properties without extrusion.

Items	1#	2#	3#	4#	5#	6#	7#
*t_10_*/min	2.34	5.42	6.31	5.73	4.34	4.09	3.00
*t_90_*/min	7.99	16.61	16.41	15.23	13.09	13.33	10.67
*M_L_*/(dN·m)	3.50	3.08	3.33	2.91	3.88	4.19	3.09
*M_H_*/(dN·m)	15.81	14.51	12.88	12.37	16.24	16.69	13.31
*(M_H_-M_L_)*/(dN·m)	12.31	11.43	9.55	9.46	12.36	12.5	10.22
Mooney *ML* (1 + 4)	76.25	76.73	78.65	74.43	87.57	97.09	77.99

**Table 3:** Results data of experiments for vulcanizate properties after extrusion.

Items	1#	2#	3#	4#	5#	6#	7#
*t_10_*/min	1.87	3.55	4.55	3.70	3.92	4.31	0.93
*t_90_*/min	6.95	13.71	13.92	13.42	11.24	12.55	7.59
*M_L_*/(dN·m)	2.79	2.40	2.63	2.18	3.16	3.31	2.59
*M_H_*/(dN·m)	14.42	12.82	12.55	12.92	15.12	15.48	10.65
*(M_H_-M_L_)*/(dN·m)	11.63	10.42	9.92	10.74	11.96	11.17	8.06
Mooney *ML* (1 + 4)	73.14	73.95	74.43	66.52	85.28	94.11	74.96

**Table 4:** Results data of experiments for vulcanizate properties without extrusion.

Items	1#	2#	3#	4#	5#	6#	7#
Hardness	62	67	65	67.5	68.5	67.5	66
σ_1_	2.43	1.41	1.28	0.87	1.49	2.60	1.41
σ_2_	19.05	19.41	19.72	19.82	21.42	20.70	21.61
ε_t_	460.8	570.4	604.6	543.0	634.3	468.9	615.7
τ_t_	92	81	71	83	88	84	85
△V	153.68	161.51	132.92	119.70	111.38	116.51	106.83
Mooney	76.25	76.73	78.65	74.43	87.57	97.09	81.68
Carbon black dispersion	5.43	6.25	6.12	6.83	6.20	6.36	6.91

In the table: ε_t_—elongation at break, %; σ_1_—100% constant elongation stress, MPa; σ_2_—tensile strength, MPa; τ_t_—tear strength, kN/m; △V—DIN wear volume, mm^3^; hardness—RT Shore A.

**Table 5:** Results data of experiments for vulcanizate properties after extrusion.

Items	1#	2#	3#	4#	5#	6#	7#
Hardness	66	62.5	64	62.5	67	67	64
σ_1_	2.35	1.25	1.56	1.63	3.01	1.81	1.41
σ_2_	20.99	18.59	19.75	20.58	22.32	21.09	22.79
ε_t_	511.6	555.8	545.5	543.0	520.7	508.4	538.8
τ_t_	90	84	79	87	83	86	86
△V	141.62	156.55	127.90	112.74	101.26	106.93	97.77
Mooney	73.14	73.95	74.43	66.52	85.28	94.11	77.99
Carbon black dispersion	7.51	7.36	7.50	7.97	7.00	7.25	7.98

## Data Availability

Not applicable.
